# MEK Inhibition Sensitizes Precursor B-Cell Acute Lymphoblastic Leukemia (B-ALL) Cells to Dexamethasone through Modulation of mTOR Activity and Stimulation of Autophagy

**DOI:** 10.1371/journal.pone.0155893

**Published:** 2016-05-19

**Authors:** Anna Polak, Przemysław Kiliszek, Tomasz Sewastianik, Maciej Szydłowski, Ewa Jabłońska, Emilia Białopiotrowicz, Patryk Górniak, Sergiusz Markowicz, Eliza Nowak, Monika A. Grygorowicz, Monika Prochorec-Sobieszek, Dominika Nowis, Jakub Gołąb, Sebastian Giebel, Ewa Lech-Marańda, Krzysztof Warzocha, Przemysław Juszczyński

**Affiliations:** 1 Dept. of Experimental Hematology, Institute of Hematology and Transfusion Medicine, Warsaw, Poland; 2 Dept. of Diagnostic Hematology, Institute of Hematology and Transfusion Medicine, Warsaw, Poland; 3 Dept. of Immunology, Maria Sklodowska-Curie Memorial Cancer Center–Institute of Oncology, Warsaw, Poland; 4 Genomic Medicine, Dept. of General, Transplant and Liver Surgery, Medical University of Warsaw, Warsaw, Poland; 5 Laboratory of Experimental Medicine, Centre of New Technologies, University of Warsaw, Warsaw, Poland; 6 Dept. of Immunology, Center of Biostructure Research, Medical University of Warsaw, Warsaw, Poland; 7 Dept. of Bone Marrow Transplantation and Hematology-Oncology, Maria Sklodowska-Curie Memorial Cancer Center and Institute of Oncology, Gliwice Branch, Gliwice, Poland; 8 Dept. of Hematology, Institute of Hematology and Transfusion Medicine, Warsaw, Poland; 9 Dept. of Hematology and Transfusion Medicine, Centre of Postgraduate Medical Education, Warsaw, Poland; Medical College of Wisconsin, UNITED STATES

## Abstract

Resistance to glucocorticosteroids (GCs) is a major adverse prognostic factor in B-ALL, but the molecular mechanisms leading to GC resistance are not completely understood. Herein, we sought to elucidate the molecular background of GC resistance in B-ALL and characterize the therapeutic potential of targeted intervention in these mechanisms. Using exploratory bioinformatic approaches, we found that resistant cells exhibited significantly higher expression of MEK/ERK (MAPK) pathway components. We found that GC-resistant ALL cell lines had markedly higher baseline activity of MEK and small-molecule MEK1/2 inhibitor selumetinib increased GCs-induced cell death. MEK inhibitor similarly increased *in vitro* dexamethasone activity in primary ALL blasts from 19 of 22 tested patients. To further confirm these observations, we overexpressed a constitutively active MEK mutant in GC-sensitive cells and found that forced MEK activity induced resistance to dexamethasone. Since recent studies highlight the role GC-induced autophagy upstream of apoptotic cell death, we assessed LC3 processing, MDC staining and GFP-LC3 relocalization in cells incubated with either DEX, SEL or combination of drugs. Unlike either drug alone, only their combination markedly increased these markers of autophagy. These changes were associated with decreased mTOR activity and blocked 4E-BP1 phosphorylation. In cells with silenced beclin-1 (BCN1), required for autophagosome formation, the synergy of DEX and SEL was markedly reduced. Taken together, we show that MEK inhibitor selumetinib enhances dexamethasone toxicity in GC-resistant B-ALL cells. The underlying mechanism of this interaction involves inhibition of mTOR signaling pathway and modulation of autophagy markers, likely reflecting induction of this process and required for cell death. Thus, our data demonstrate that modulation of MEK/ERK pathway is an attractive therapeutic strategy overcoming GC resistance in B-ALL patients.

## Introduction

Synthetic glucocorticoids (GCs) such as dexamethasone or prednisolone have been used for decades in the treatment of acute lymphoblastic leukemia (ALL) and other malignancies [1]. Current chemotherapy regimens allow achieving complete remission (CR) in the majority of ALL patients, but about 20% of children and 60% of adults eventually relapse [2–7]. *In vitro* and *in vivo* response to glucocorticoids is a major prognostic factor in childhood ALL [5, 7–9]. Resistance to glucocorticoids is much more frequent in adult ALL, and resistance to GCs is a common feature of relapsed leukemic clone [2–7]. Since lymphoblasts from children and adults who achieve CR are *in vitro* more sensitive to GCs, major efforts are being made for better understanding of the molecular mechanisms driving resistance to these drugs. This knowledge might be an important step toward development of targeted therapeutic strategies restoring drug sensitivity, reducing risk of relapse and thus, improving patients outcome.

Despite their extensive use and tremendous clinical impact, the mechanisms by which GCs exert their biological and clinical effects are incompletely understood. GCs action within the cells is initiated upon binding to the glucocorticoid receptor (GR), responsible for the induction of genomic and non-genomic effects. Treatment with GCs in leukemic cells leads to G1 phase cell cycle arrest and induction of a programmed cell death (apoptosis). Multiple intermediate pathways and mechanisms have been implicated in mediating these effects; likewise, many mechanisms have been identified to contribute to GC-resistance, including certain protein kinases (e.g. GSK3, AKT, mTOR, AMPK), expression of BCL2-family members (MCL1, BCL-XL), activity of deubiquitinase USP9X, or posttranslational modifications of FOXO3a [10–14].

Apoptosis has been suggested to be the main effector mechanism associated with GCs therapy [13, 15, 16], but recent studies highlighted the role of autophagy upstream of apoptotic cell death [17–19]. Autophagy is a highly conserved process, regulating normal protein and organelle turnover, characterized by the formation of double-membrane vesicles, called autophagosomes, that engulf a portion of the cytoplasm and deliver it for lysosomal degradation [20]. The formation of autophagic vesicle depends on a class III phosphatidylinositol 3-kinase PI3K, beclin-1, and is inhibited by the AKT/mTOR pathway in response to various growth factors [21, 22]. Although autophagy was initially described as a process that facilitates cellular survival under starvation or metabolic stress, it may also lead to cell death, presumably by an excessive degradation of cellular components [23, 24].

In this study, we examined potential mechanisms responsible for glucocorticoid resistance in ALL cells, and found that blasts resistant to GCs exhibit significantly higher expression of mitogen-activated protein kinase (MAPK/ERK) pathway components. We show that MEK1/2 inhibitor selumetinib enhances DEX toxicity in GC-resistant B-ALL cells. The mechanism underlying this interaction involves inhibition of mTOR signaling pathway, induction of certain markers of autophagy and subsequent cell death. These observations provide new insights into the biological bases of clinically observed GCs resistance and demonstrate that modulation of autophagy by MEK/ERK pathway is a new, targetable mechanism of GC resistance in B-ALL patients. From the clinical standpoint, these observations may facilitate development of clinical trials testing optimized treatment protocols and maximizing chances for complete remission and cure of ALL patients.

## Materials and Methods

### Bioinformatics and statistical analyses

To identify potential mechanisms responsible for GC-resistant phenotype, we utilized gene expression data from previous studies that assessed transcriptional profiles of GC-sensitive and GC-resistant cells [25, 26]. Primary data deposited under ID GSE5820 and GSE32962 are available from Gene Expression Omnibus, (http://www.ncbi.nlm.nih.gov/geo/). We used these data to perform an exploratory *in silico* data mining approach using Gene Set Enrichment Analysis (GSEA) www.broadinstitute.org/gsea/. Top genes differentiating studied phenotypes were ranked with respect to the absolute signal to noise ratio (SNR) [27, 28] and subsequently interrogated with gene sets from the MSigDB repository (representing e.g. a common signaling pathways). The proximity of a given gene set to the top end of the ranked list were measured with an enrichment score (ES) and Kolmogorov-Smirnov [29] statistics and by comparing the observed ES to the distribution of permuted ES scores [30–32].

Data are presented as means from three independent experiments ± SD, unless otherwise stated. Statistical comparisons were performed with Student’s *t*-test. P-values <0.05 were considered statistically significant. All calculations were performed using GraphPad Prism or R software.

### Cell lines and primary ALL blasts

Human pre-B ALL cell lines SEMK2, RS4;11, 697 and a T-ALL cell line CCRF-CEM were obtained from DSMZ (Braunschweig, Germany). Cell lines were maintained at a density of 0.6–2.0×10^6^/ml in RPMI-1640 medium (Lonza) supplemented with 10% heat inactivated fetal bovine serum (Biovest), 2 mM L-glutamine, 10 mM HEPES and 100 U/ml of penicillin/streptomycin (Lonza). Peripheral blasts from adult pre-B-ALL patients were isolated using density gradient centrifugation (Ficoll-Hypaque; density 1.077 g/mL) within 24 hours from harvesting. If necessary, ALL samples were further purified to achieve more than 90% blasts by removing nonmalignant cells using immunomagnetic beads (B-cell isolation kit, Miltenyi). Cells were then frozen in liquid nitrogen or cultured at 2×10^6^/ml concentration in RPMI-1640 medium (Lonza) containing 20% heat-inactivated fetal bovine serum (FBS), 2mM L-glutamine, 10mM HEPES and 100U/mL of penicillin/streptomycin. The study was conducted after approval of Bioethics Committee (Komisja Bioetyczna, http://www.ihit.waw.pl/komisja-bioetyczna.html). The study was conducted according to Declaration of Helsinki principles. Patients provided written informed consent, and all sensitive information were blinded and data were analyzed anonymously.

### Chemicals, antibodies and vectors

Dexamethasone was used at 0.05 μg/ml (0.1 μM), 2 μg/ml (4 μM) or 30 μg/ml (60 μM) concentrations, as indicated. MEK1/2 inhibitor selumetinib (Selleck Chem) was used at 200 nM, chloroquine (Sigma Aldrich) was used at a 50 μM or 100 μM and monodansylcadaverine (MDC) (Sigma Aldrich) at 50 μM concentrations, mTORC1 inhibitor rapamycin at 100 nM. Selumetinib, chloroquine and MDC were dissolved in DMSO and stored at -20°C. Antibodies against LC3 A/B (#4108), BIM (#2933), p-ERK^T202/204^ (#4370), p4E-BP1^T37/42^ (#5114), p-p90RSK^T353/S356^ (#9344), 4E-BP1 (#9452), BCN1 (#3738), RSK1/2/3 (#9355), GR (#3660) and β-actin (#4970) were purchased from Cell Signaling. Antibodies against GAPDH (#MAB 374) and ERK1/2 (#05–1152) were purchased from Millipore. Plasmids encoding MEK1 kinase and pBabe-puro were obtained from dr W. Hahn and dr H. Land *via* Addgene [33, 34]. Introduction of Q56P mutation into MEK1 sequence was performed using two-stage site directed PCR mutagenesis followed by fusion PCR. In a first stage of mutagenesis N- and C-termini of MEK1 kinase were amplified with primers introducing desired mutation. Both fragments overlapped in 25 base pairs. In the second stage of mutagenesis, products of the first stage PCR were used as a templates to perform fusion PCR with flanking primers. Primers sequences are given in Table 1. pLVX-IRES-PURO-GFP-LC3 plasmid containing rat LC3 protein N-terminally fused to GFP was generated from a pEGFP-LC3m plasmid (a kind gift of Prof. Noboru Mizushima and Dr Tamotsu Yoshimori [35]) using XhoI and NotI sites. All constructs were sequenced and confirmed to be correct. Vectors were then used to transform XL1 blue bacterial strains and plasmid DNA was prepared and purified using an Endo-free Plasmid maxi kit (Qiagen), according to the manufacturer’s instructions. Plasmids were dissolved in endotoxin-free TE buffer and stored at −20°C until use. Vectors were used to generate retroviruses and infect target cells as previously described [36].

**Table 1:** Primer sequences used for MEK1 mutagenesis.

Name	Primer sequence
**MEKQ56P_BamHI_F**	**5’ CTTTGTACACCCTAAGCCTCCG 3’**
**MEKQ56P_SnaBI_R**	**5’ TAGATACGTAGCGGCCGCCTAGATG 3’**
**Q56PI_R**	**5’CTTCTGCTTCGGCGTCAGAAAGGCC 3’**
**Q56PII_F**	**5’GGCCTTTCTGACGCCGAAGCAGAAG 3’**

### shRNA-mediated BCN1 downregulation

Short hairpin (sh) RNA targeting BCN1 gene was designed as previously described [37]. Single-stranded shRNA’s corresponding to the 5’GTAAGACGTCCAACAACAGC3’ sequence in BCN1 cDNA were purchased from Sigma-Aldrich and annealed. Obtained double-stranded oligo was cloned into pSIREN-RetroQ-Puro retroviral vector (Clontech) using BamHI/EcoRI sites as described [36] and retrovirally transferred to SEMK2 cells. BCN1 silencing was assessed by immunoblotting.

### Cell death assay

Cell death was measured using AnnexinV—fluorescein isothiocyanate (FITC) and propidium iodide (PI) staining according to manufacturer instructions (Annexin V—FITC apoptosis detection kit I, BD Biosciences). Cell death data were acquired on a BD FACS Canto I flow cytometer (BD Biosciences) and analyzed by FacsDiva software (BD Biosciences).

### Immunoblotting

Cells were washed in PBS and lysed in RIPA buffer supplemented with protease and phosphatase inhibitor cocktail (Complete Protease Inhibitor Cocktail Tablets, PhosSTOP Phosphatase Inhibitor Cocktail Tablets, Roche) as described [38]. Proteins were size-fractionated by sodium dodecyl sulfate (SDS)–PAGE and transferred to Immunobilon-PVDF membranes (Millipore). Blots were incubated in blocking buffer (5% BSA, 0.1% Tween/Tris-buffered saline TBS) at room temperature for 1 hour and subsequently incubated with primary antibodies diluted 1:1000 in 5% BSA/TBST overnight at 4°C with rotation. After washing in TBST, blots were incubated with appropriate horseradish peroxidase (HRP)–conjugated secondary antibodies at room temperature for 1 hour, developed by ECL (Perkin Elmer), and visualized with G:Box image acquisition system (Syngene). To reprobe with another antibody, blots were incubated in stripping buffer at 50°C for 30 minutes and analyzed as described above. Densitometric analyses were performed using ImageJ software (www.imagej.net).

### Phospho-specific flow cytometry

Activity of MAPK/ERK pathway in primary blasts was assessed using intracellular phospho-specific flow cytometry according to manufacturer’s protocol (BD Phosflow Protocol II for Human PBMCs, BD Biosciences). Briefly, 1×10^6^ cells were resuspended in 1 mL of ice cold PBS, then fixed, permeabilized and stained with phycoerythrin conjugated pERK1/2 (pT202/pY204) or isotype control antibody (IgG1ĸ) (BD Bioscience). Flow cytometric analysis was performed on FACS Canto I flow cytometer (BD Bioscience) and analyzed by Facs Diva.

### Monodansylcadaverine staining (MDC) and fluorescence microscopy

For labeling and visualization of MDC labeled vacuoles, SEMK2 and RS4;11 cells (2 × 10^5^) were incubated with DEX, SEL or combination of drugs for 24h, then stained with MDC (50 μM) for 15 minutes at 37°C in the dark and washed 3 times with PBS, centrifuged with a cytospin centrifuge and directly visualized by a fluorescence microscope, equipped with DAPI filter and CCD camera (Axio Imager.Z2, Zeiss). SEMK2 cells stably expressing GFP-LC3 were washed with PBS, stained with Hoechst 33342 (2.5 μg/ml) (Life technologies) for 20 minutes in the dark, again washed 3 times with PBS, centrifuged with a cytospin centrifuge and directly visualized using Axio Imager.Z2 fluorescence microscope equipped with FITC/DAPI filters and CCD camera. All fluorescence images were captured at 630 × magnification.

## Results

### MAPK/ERK gene expression signature and activity differentiates GC-resistant and GC-sensitive ALL blasts

**Fig 1:**
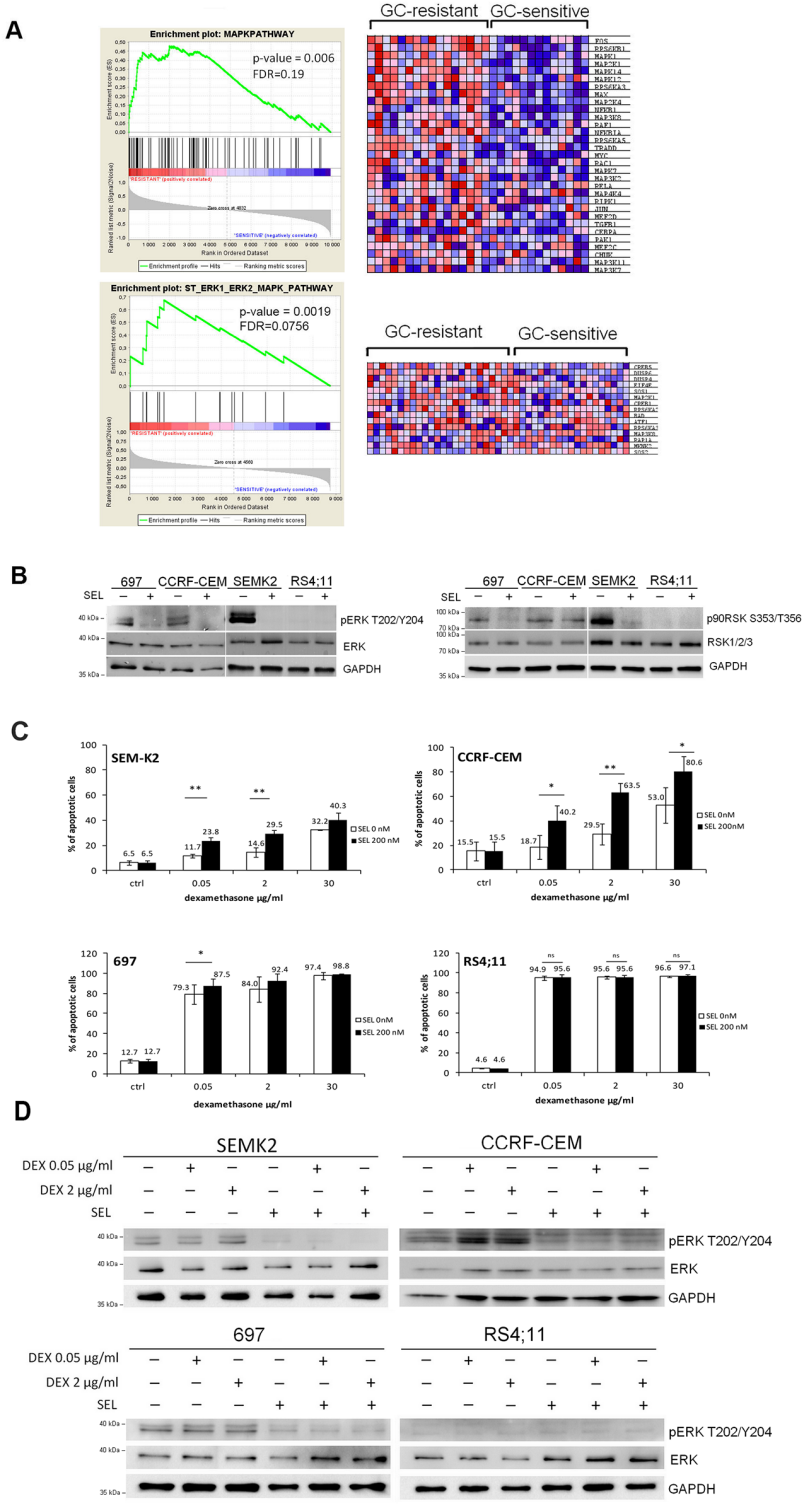
MEK1/2 inhibitor, selumetinib sensitizes GC-resistant ALL cells to dexamethasone. (A) GC-resistant cells exhibit coordinate upregulation of MAPK/ERK pathway components. GSEA plots show relative upregulation of MAPK/ERK cascade components in GC-resistant ALL cells in two independent datasets [25, 26]. Relative expression of pathway components is visualized by the heat map. FDR- false discovery rate. (B) ALL cell lines with active MAPK/ERK pathway (SEMK2, 697, CCRF-CEM) and ALL cells with undetectable expression of MAPK/ERK pathway (RS4;11) were incubated for 4h with MEK1/2 inhibitor, selumetinib (SEL, 200 nM). Thereafter, phosphorylation status of ERK1/2 and its substrate p90RSK were assessed by immunoblotting. (C) ALL cell lines were incubated with DEX (0.05 μg/ml 2 μg/ml and 30 μg/ml), SEL (200 nM) or combination of DEX+SEL for 72h and cell death was assessed by annexinV/PI staining followed by flow cytometry analysis. * p<0.05, ** p<0.01, ns- not significant. Error bars represent SD of three independent experiment. P values were calculated using Student’s t-test. (D) Cells were incubated as in (C) and used to determine the phosphorylation status of ERK1/2 by immunoblotting.

To elucidate the molecular mechanisms responsible for GCs resistance in B-ALL blasts, we performed gene set enrichment analysis of publicly available gene expression profiles obtained from GC-resistant and -sensitive primary B-ALL blasts [25, 26]. These analyses revealed that GC-resistant cells exhibit significantly higher expression of MAPK/ERK pathway components as compared to sensitive cells (p = .006, FDR = 0.19 and p = .0019, FDR = 0.0756 (Fig 1A). These observations led us to hypothesize that MAPK/ERK activation might contribute to cellular resistance to GCs. To test this hypothesis, we determined the activation status of MAPK/ERK pathway in ALL cell lines. Since ERK1/2 is the only physiological substrate of MEK1/2, the activity of MAPK/ERK pathway can be monitored by evaluating the level of phosphorylated ERK^T202/204^ and its substrate, p90RSK^S353/T356^. Three of four examined ALL cell lines (SEMK2, 697, CCRF-CEM) exhibited baseline phosphorylation of ERK1/2 and p90RSK, which rapidly and markedly decreased after incubation with MEK1/2 inhibitor, selumetinib (SEL), approved for treatment of melanoma and tested in other solid malignancies (Fig 1B). To assess whether inhibition of MAPK/ERK pathway would sensitize ALL cells to dexamethasone, we incubated cells with high (SEMK2, 697, CCRF-CEM) or undetectable (RS4;11) activity of MAPK/ERK pathway with increasing concentrations of dexamethasone (DEX), selumetinib (SEL) or combination of these drugs (DEX+SEL) for 72h. Cells were subsequently stained with annexinV/PI and cell death was assessed by flow cytometry analysis. Inhibition of MEK1/2 increased dexamethasone toxicity in all resistant cell lines (Fig 1C). Of note, DEX alone in resistant cells did not change the pERK levels (Fig 1D).

**Fig 2:**
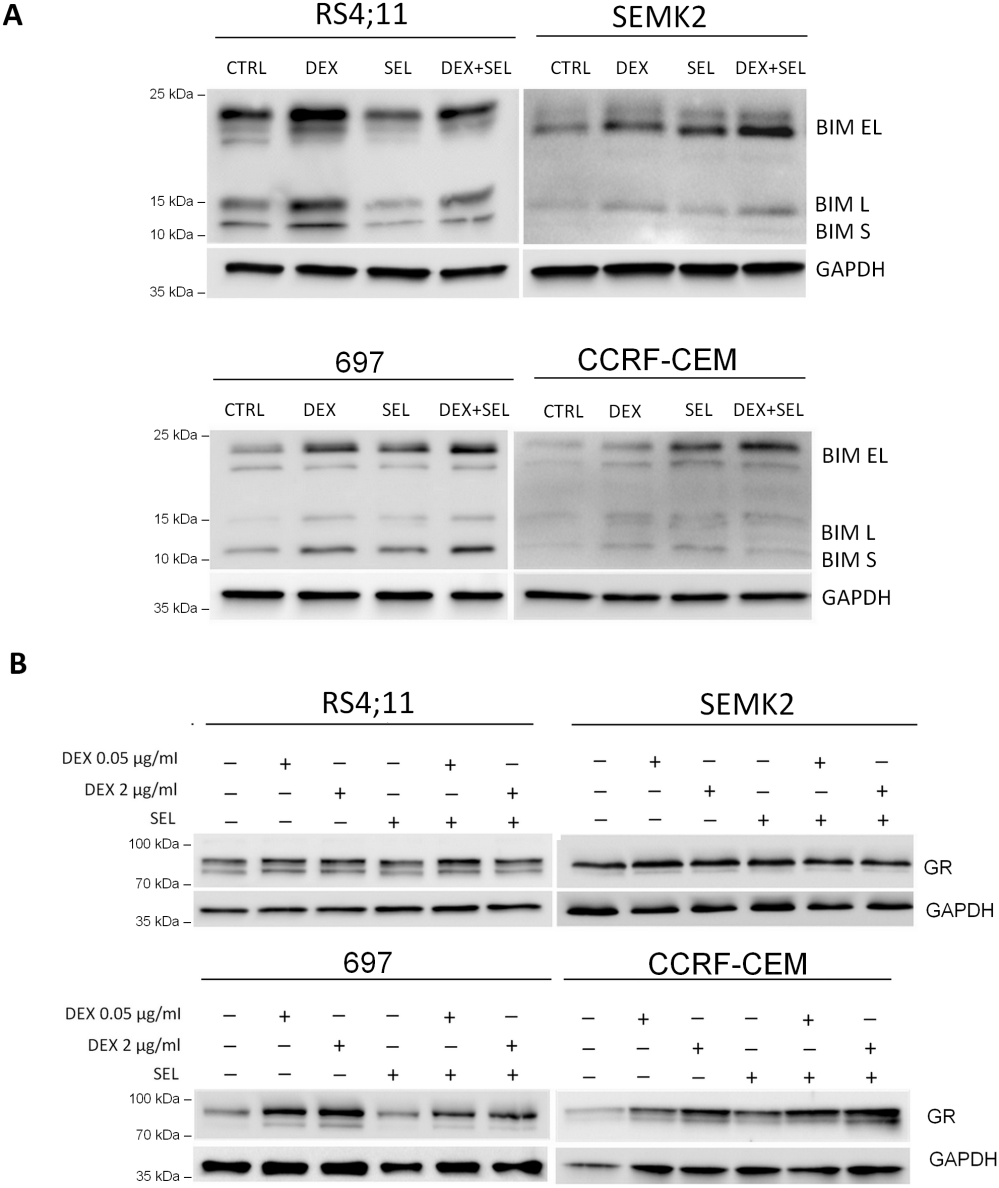
BIM and GR expression in cells incubated with DEX, SEL or their combination. GC- resistant and -sensitive SEMK2 and RS4;11 were incubated for 24 h with DEX (0.05–2 μg/ml), SEL (200 nM) or combination of DEX+SEL. BIM (A) and GR (B) expression levels were assessed by western blot.

**Fig 3:**
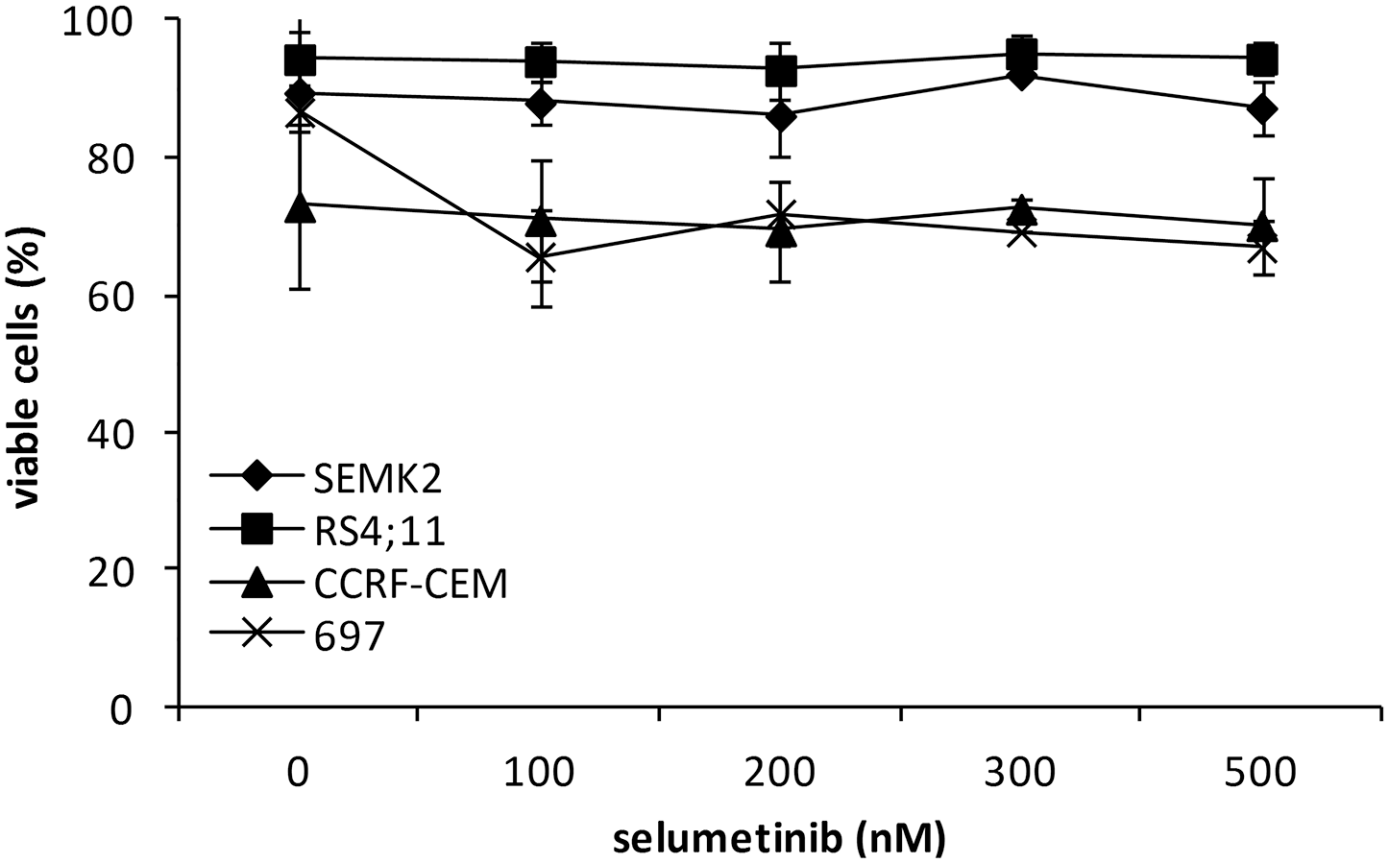
Viability of ALL cell lines incubated with SEL. ALL cell lines were incubated with SEL at the indicated doses for 72h and cell death was assessed by annexinV/PI staining followed by flow cytometry analysis. Error bars indicate SD from at least 3 independent repeats.

In line with these observations, DEX triggered a marked induction of a BH3-only proapoptotic BCL2 family member, BCL2L11 (BIM) in sensitive RS4;11 cells. In contrast, in GC-resistant SEMK2, CCRF-CEM and 697 cells, BIM abundance was increased only when DEX was combined with SEL (Fig 2A). DEX-sensitive and—resistant cells exhibited similar baseline levels of glucocorticosteroid receptor (GR), indicating that the baseline level is not an important determinant of apoptotic response (Fig 2B). Importantly, SEL used as a single agent did not exhibit significant toxicity to ALL cell lines (Fig 3), indicating that MEK activity is not required for cell survival.

### Overexpression of a constitutively active MEK1 mutant in GC-sensitive cells (RS4;11) induces resistance to GCs

**Fig 4:**
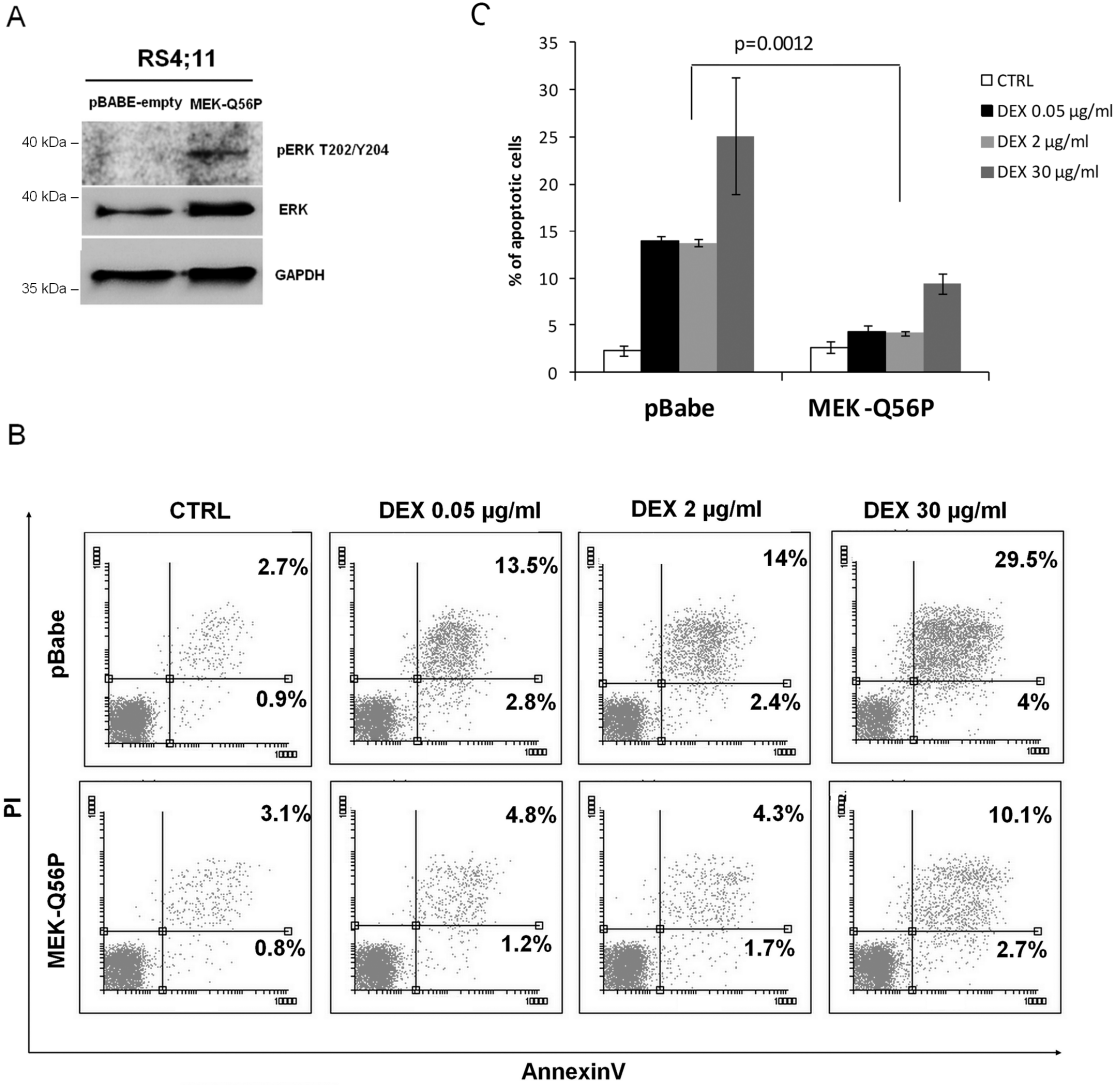
Overexpression of a constitutively active MEK1 mutant (MEK-Q56P) in GC-sensitive RS4;11 cells induces resistance to DEX. (A) RS4;11 cells were retrovirally transduced with MEK-Q56P or empty control. Cells were lysed and ERK1/2 phoshorylation status was assessed by immunoblotting. (B-C) Control cells and MEK-Q56P—transduced cells were incubated with DEX (0.05, 2 or 30 μg/ml) for 72h. Thereafter, cell death was assessed by annexinV/PI staining and flow cytometry analysis. Absolute, averaged numbers of apoptotic cells in two independent experiments are indicated in (C). Error bars represent SD. P value was calculated using Student’s t-test.

To further evaluate the role of MAPK/ERK pathway in mediating GCs resistance, we overexpressed constitutively active form of MEK1 kinase (MEK-Q56P) in DEX-sensitive RS4;11 cells, which do not exhibit detectable activity of MAPK/ERK pathway. MEK-Q56P mutation, identified in melanoma patients, is located in the proximity of regulatory helix A and leads to constitutive kinase activity [39]. Compared to control RS4;11 cells transduced with the empty vector (pBabe), cells transduced with this MEK1 mutant exhibited increased basal ERK1/2 activation (Fig 4A). Consistent with our previous results, MEK-Q56-transduced cells exhibited substantially lower sensitivity to dexamethasone-induced cell death than control cells (Fig 4B and 4C).

### MEK1/2 inhibitor, selumetinib, potentiates DEX-induced LC3 processing, MDC staining and GFP-LC3 relocalization in ALL cells

**Fig 5:**
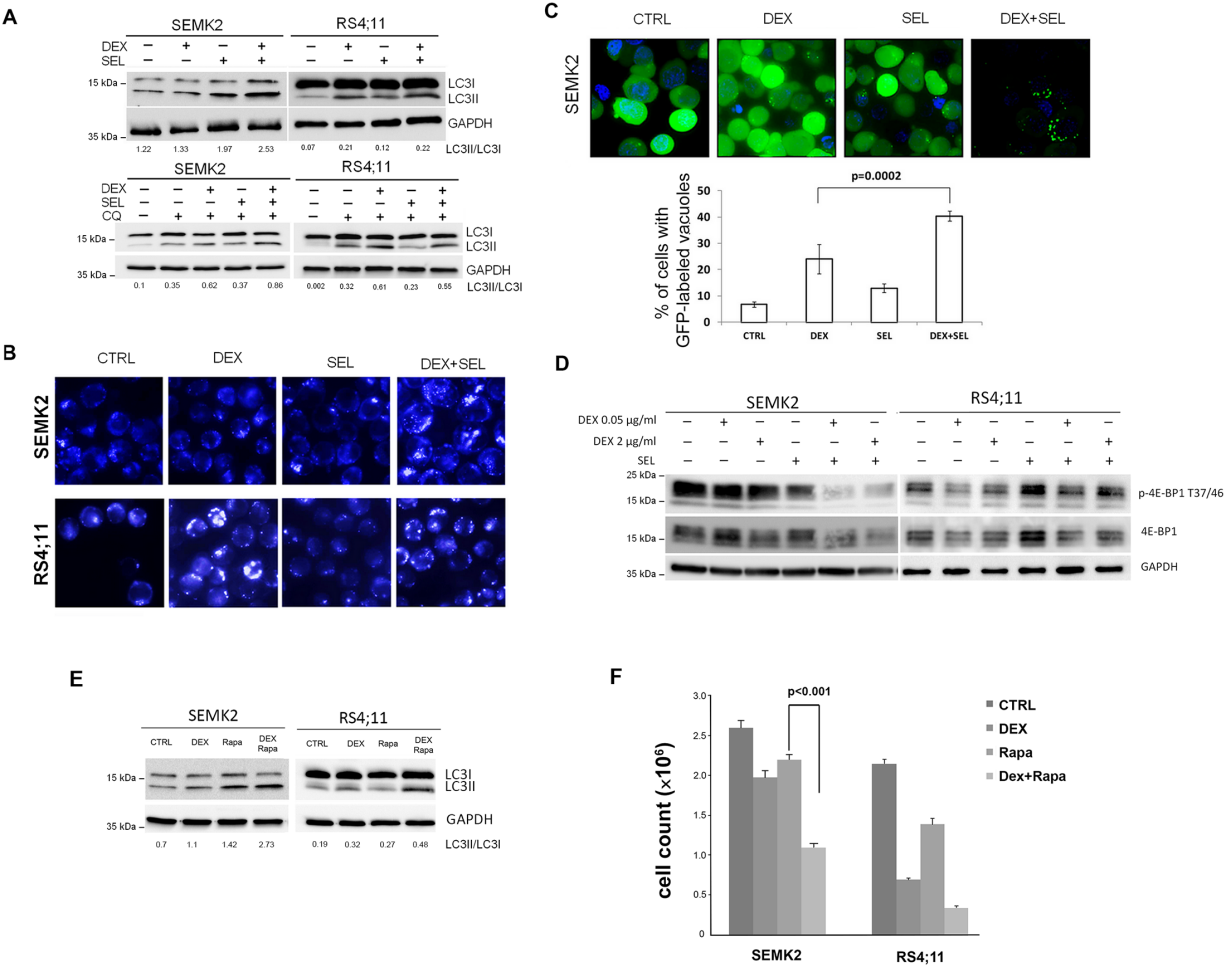
MEK1/2 inhibitor, selumetinib, intensifies DEX induced LC3 conversion, MDC staining and GFP-LC3 relocalization in GC-resistant SEMK2 ALL cells. (A) GC-sensitive (RS4;11) and GC-resistant (SEMK2) cells were incubated with DEX (0.05 μg/mL) in the presence or absence of MEK1/2 inhibitor, selumetinib (SEL, 200 nM) for 24h. When indicated, cells were pretreated for 3 h with 50 μM or 100 μM of chloroquine (CQ). Thereafter, LC3 processing was assessed by immunoblotting. Densitometric analyses of LC3II/I are indicated below the blots. (B) SEMK2 and RS4;11 cells were cultured as described above for 24h, stained with MDC (50 μM) and analyzed by fluorescence microscopy. (C) SEMK2 cells were stably transduced with GFP-LC3 and incubated with DEX, SEL or combination of DEX+SEL for 24h. GFP-LC3 relocalization from diffuse cytoplasmic in control cells to a massive dotty pattern in DEX+SEL treated cells indicates LC3 recruitment to autophagosome membranes. In the lower panel, the percentage of cells with GFP-LC3 dots was quantified by counting the number of cells with > 3 dots and divided by a total number of GFP positive cells in 5 random non-overlapping fields. Pictures were taken at 630 × magnification. P value was calculated using Student’s t-test. (D) Induction of autophagy markers by the DEX and SEL co-treatment involves mTOR suppression. SEMK2 and RS4;11 cells were incubated with DEX in the presence or absence of SEL and lysed. 4E-BP1 phoshorylation status was assessed by immunoblotting. (E) GC-resistant (SEMK2) and—sensitive (RS4;11) cells were incubated with mTOR inhibitor rapamycin (100nM) in the presence or absence of DEX (0.05 μg/mL) for 24h. Thereafter, LC3 processing was assessed by immunoblotting and quantified. Densitometric analyses of LC3II/I are indicated below the blots. (F) Cells were treated as in (E) for 72h. Thereafter, cell numbers were assessed by counting 6 independent fields in Burker’s chamber. Data represent two independent experiments. P-values were calculated using 2-sided Student’s t-test.

We next sought to identify molecular mechanisms responsible for the mechanisms of MEK1/2 inhibitor—induced sensitization of ALL cells to DEX. Since in GC-sensitive cells treatment with DEX leads to induction of autophagy that precedes apoptotic cell death [17], we hypothesized that MAPK/ERK pathway contributes to GC-resistance by suppressing DEX-induced autophagy. To test this hypothesis, we incubated GC-resistant and -sensitive ALL cell lines with DEX, SEL or their combination for 24h and then assessed three independent markers of autophagy. First, we assessed conversion of microtubule-associated protein light chain 3 (LC3-I) to LC3-II. During the formation of autophagosomes in mammalian cells, carboxyl-terminal region of LC3 is cleaved generating a soluble form LC3-I. LC3-I is subsequently modified to a membrane-bound form LC3-II, localized to autophagosomes and autophagolysosomes. Therefore, conversion of LC3-I to LC3-II is widely used to monitor autophagy [35, 40, 41]. We found that in DEX-sensitive RS4;11 cells, DEX induced conversion of LC3-I to LC3-II, which was not further increased by co-administration of DEX/SEL (Fig 5A). In GC-resistant SEMK2 cells, DEX or SEL alone had a noticeable effect on the LC3-II abundance, but the simultaneous incubation with DEX and SEL resulted in markedly increased LC3-II levels (Fig 5A).

While most of the LC3-II at the outer autophagosomal membrane is recycled to the cytosol, the fraction of LC3-II at the inner membrane is transported to the lysosomal compartment where it is degraded. Thus, it is important to perform LC3 turnover assays comparing the levels of LC3-II in the presence of lysosomal inhibitors, like chloroquine (CQ), that prevent degradation of autophagic vacuoles by increasing lysosomal pH [40, 42]. Incubation of the RS4;11 cells with CQ alone increased the amount of LC3-II form, reflecting basal level of autophagy in these cells (Fig 5A). Incubation of these cells with DEX and CQ led to increased amounts of LC3-II above the levels induced by either CQ or DEX alone (Fig 5A). In GC-resistant SEMK2 cells, co-cultured with DEX or SEL in the presence of CQ inhibitor, the abundance of LC3-II form moderately increased, but cells incubated with DEX and SEL simultaneously exhibited markedly greater increase in LC3-II form compared to cells incubated with DEX or SEL alone (Fig 5A).

To further assess the putative role of autophagy in cell death induced by DEX and SEL, we stained these cells with monodansylcadaverine (MDC), a dye of autophagosomes and autophagolysosomes [42]. The amount of MDC-positive structures in RS4;11 cells increased after DEX and did not change in response to SEL or combination of DEX/SEL. In contrast, in DEX-resistant SEMK2 cells, the number of MDC-positive structures in response to DEX or SEL alone did not change significantly as compared to control cells, but combination of these agents markedly increased the abundance of MDC-positive structures (Fig 5B).

Because MDC is not a specific marker of autophagy, as it associates not only with acidic (lysosomes, autophagolysosomes) but also with lipid-rich compartments (autophagic vacuoles and lamellar bodies), we further performed fluorescence-based visualization of autophagosomes using LC3 N-terminally fused to GFP (GFP-LC3). This approach allows to monitor autophagy by observing LC3 protein relocalization from a diffuse cytoplasmic to a dotty pattern, indicating recruitment of this protein to autophagosome membranes [42, 43]. For this purpose, we retrovirally transduced DEX-resistant SEMK2 cells with pMIP-GFP-LC3 vector. After obtaining stable transfectants, cells were incubated with DEX, SEL or combination of these drugs for 24 h. Consistent with previous results, DEX or SEL treatment alone induced only weak GFP-LC3 relocalization to autophagosome membranes in GFP-positive cells, but co-administration of this drugs robustly enhanced LC3 relocalization to a distinct punctual pattern (Fig 5C).

### Combination of MEK1/2 inhibitor with DEX in resistant cells inhibits mTOR activity

Autophagy is controlled by mTOR, thought to be a master-regulator of this process [44]. Reduction of mTOR activity is associated with induction of autophagy. We hypothesized that constitutive MEK activity in ALL cells might suppress DEX-mediated induction of autophagy by modulating mTOR. Of note, our previous data indicated that MEK1/2 inhibition led to decreased activity of ERK1/2 and p90RSK (Fig 1B). Both ERK1/2 and p90RSK phosphorylate and inactivate TSC2, functioning as a molecular switch that regulates mTOR’s activity [45–47]. To test this hypothesis, we assessed the activity of a key mTORC1 substrate, 4E-BP1, in DEX-resistant and DEX-sensitive ALL cell lines following incubation with DEX, SEL or both compounds simultaneously. In RS4;11 cells DEX, but not SEL, decreased the level of phosphorylated of 4E-BP1 (Fig 5D). In contrast, in SEMK2 cells, incubation with DEX or SEL alone only moderately changed the phosphorylation level of 4E-BP1, but co-treatment resulted in a marked inhibition of this protein (Fig 5D). Consistent with these findings, an mTORC1 inhibitor rapamycin similarly synergized with DEX (Fig 5E and 5F) with respect to induction LC3 conversion and inhibition of cellular proliferation. Thus, mTOR activation downstream of MAPK/ERK signaling pathway plays an important role in inducing resistance to GCs in ALL blasts.

### BCN1 is required for MEK1/2 inhibitor-dependent sensitization of ALL cells to DEX

**Fig 6:**
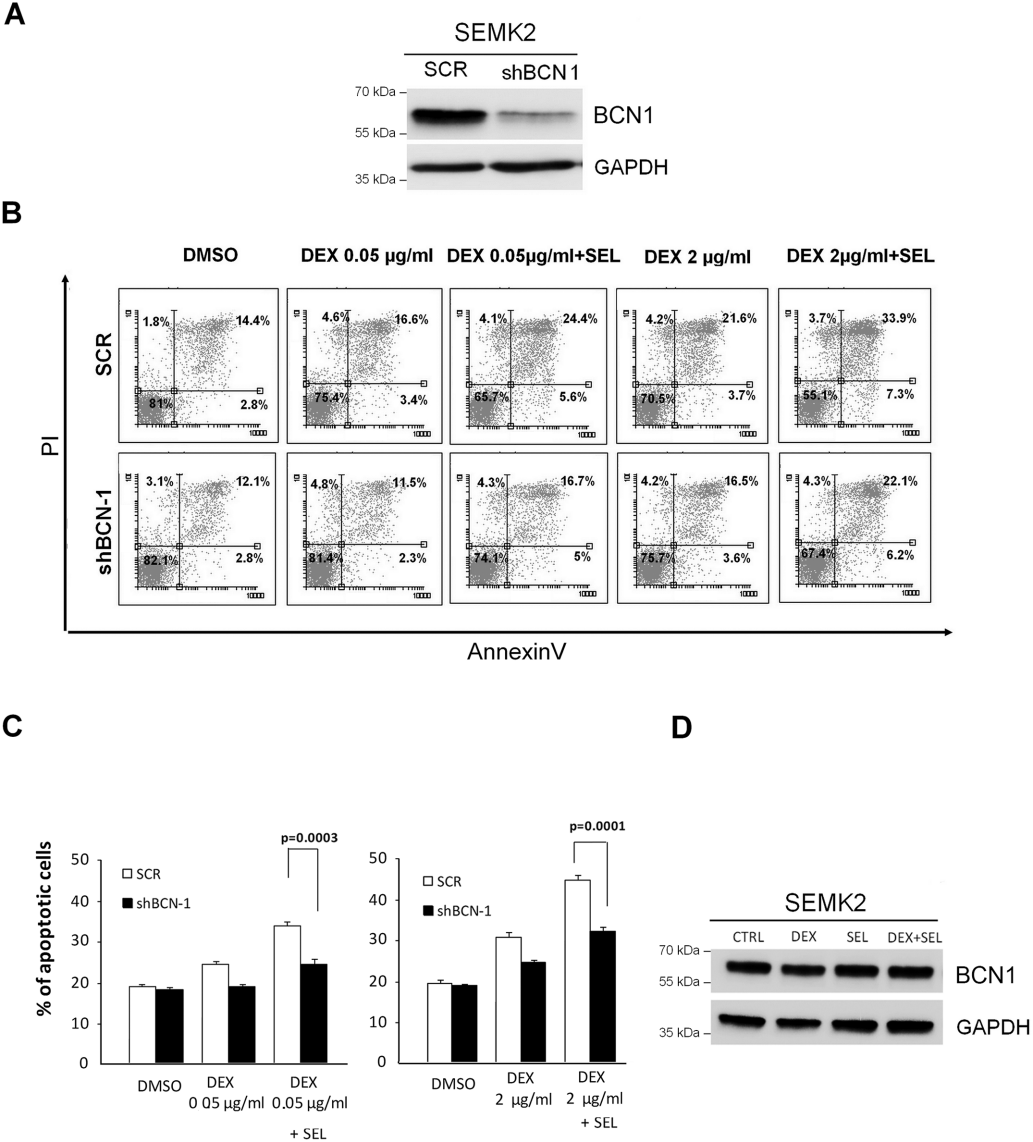
BCN1 knockdown reduces SEL-mediated sensitization to DEX. (A) SEMK2 cells were retrovirally transduced with BCN1-specific shRNA or scrambled control (SCR). After selection of stable transfectants, BCN1 knockdown was confirmed by western blot. (B,C) Cells with BCN1 knockdown or control cells were incubated with DEX, (0.05 μg/ml or 2 μg/ml) in the presence or absence of MEK1/2 inhibitor, selumetinib (SEL, 200 nM) for 72h. Apoptosis was assessed by annexinV/PI staining followed by flow cytometry analysis. Representative dot-plots from FACS analysis are shown in B; averaged results from 3 independent experiments with SD are indicated in (C) Statistical difference in responses between mock- and shBCN1-transduced cells were determined using 2-sided Student’s t-test. (D) Beclin-1 expression in cells treated with DEX, SEL or their combination. SEMK2 cells were incubated for 24 h with DEX (0.05 μg/ml), SEL (200 nM) or combination of DEX+SEL and BCN1 expression level was assessed by western blot.

After demonstrating that inhibition of MAPK/ERK pathway concurrent with GCs treatment in ALL cell lines increases autophagy and GC-induced cell death, we asked whether BCN1, a gene required for autophagosome formation, is required for this synergy. We thus compared responses to DEX/SEL treatment in SEMK2 cells with silenced BCN1, and in control cells transduced with non-targeting shRNA (Fig 6A). As expected, SEL sensitized mock-transduced cells to DEX. In contrast, BCN1-deficient cells exhibited markedly lower induction of cell death after SEL/DEX treatment (Fig 6B and 6C). Importantly, expression of this protein is not modulated by DEX or SEL treatment in ALL cells (Fig 6D). These data indicate that the increased toxicity of DEX/SEL combination in ALL cells is at least partially reliant on expression of BCN1.

### MEK1/2 inhibitor sensitizes pre-B ALL primary blasts to glucocorticoid treatment through a mechanism involving modulation of mTOR activity and induction of LC3 processing

**Fig 7:**
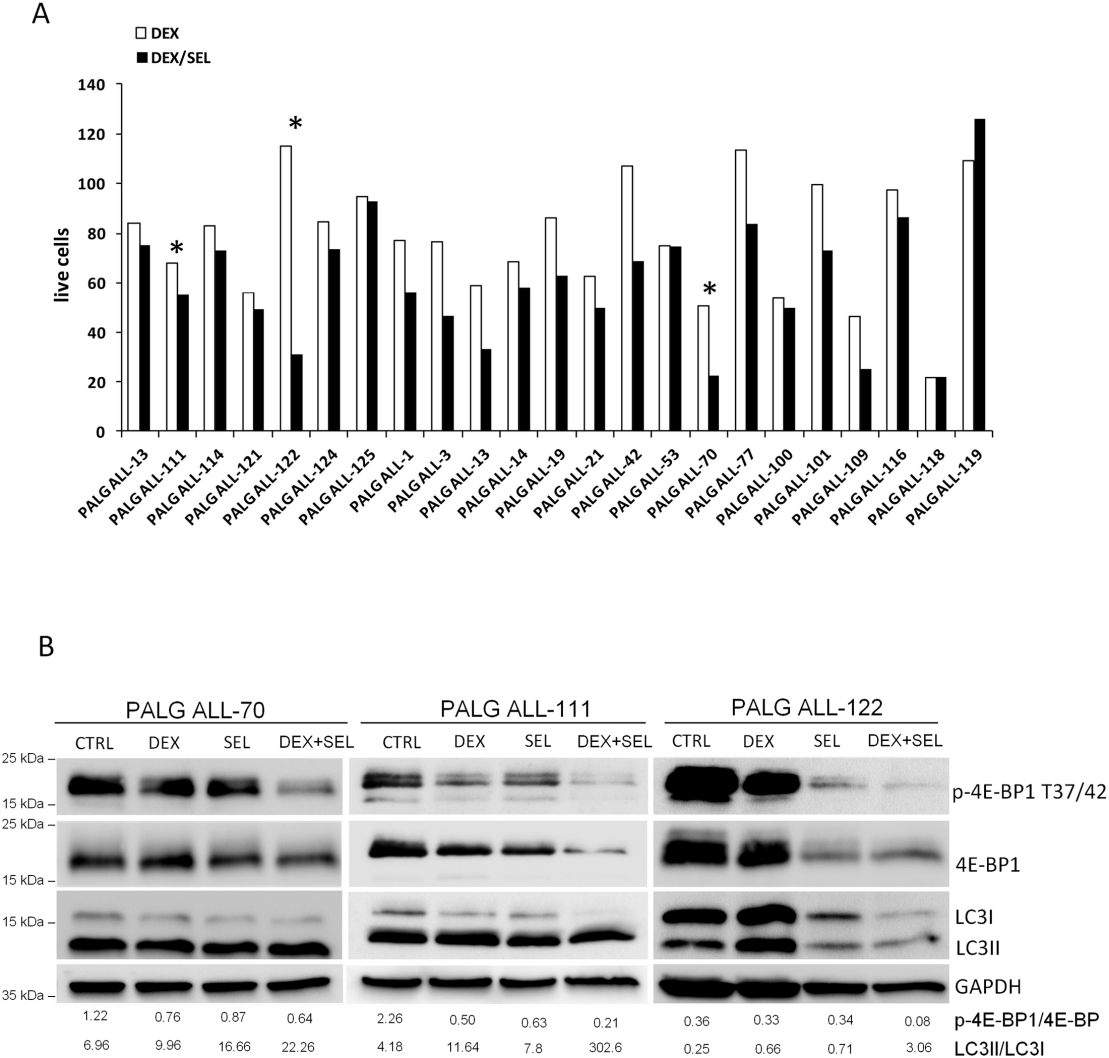
Selumetinib sensitized primary ALL blast do DEX through modulation of mTORC1 activity. (A) Primary blast were isolated from peripheral blood of ALL patients and incubated with DEX (0.05 μg/ml), SEL (200 nM) or DEX+SEL. Cell death was assessed by annexinV/PI staining and flow cytometry analysis. (B) Primary blasts from patients indicated with asterisks (panel A) were incubated as described above and phosphorylation status of 4E-BP1 and LC3 processing were assessed in total cell lysates by immunoblotting. Densitometric analyses of LC3II/I are indicated below the blots.

We next assessed responses to DEX/SEL combination in blast samples obtained from peripheral blood of 22 newly diagnosed adult ALL patients. All these samples exhibited basal ERK1/2 activity, although its magnitude varied considerably (S1 Fig). After incubation of these primary cells with DEX (0.05 μg/ml) or combination of DEX/SEL, cell death was assessed by AnnexinV/PI staining. DEX treatment alone led to variable apoptotic responses. Concurrent incubation of primary ALL blasts with DEX and SEL increased the fraction of dead cells in 17 of 22 patients by 10.2–73.3% (mean 28.6%), compared to DEX alone. In 2 patients, SEL/DEX combination exhibited only a weak effect (apoptosis increased by 2–7%) and in remaining 3 patients, SEL had no effect on DEX response (Fig 7A). We further assessed the molecular markers of autophagy in 3 representative ALL blasts specimens, in which SEL increased DEX-induced apoptosis. Combination of DEX and SEL markedly decreased phosphorylation of 4E-BP1(T37/42) (indicating decreased mTORC1 activity) and increased level of autophagy, as measured by LC3 turnover (Fig 7B). These observations indicate that in primary ALL cells with active MAPK/ERK signaling, the drugs cooperatively decrease mTOR activity and stimulate LC3 processing and relocalization, likely reflecting increased autophagy.

## Discussion

GC resistance in the initial phase of chemotherapy is a strong predictor of adverse outcome for B-ALL. Multiple studies were aimed to elucidate the mechanisms underlying defective induction of cell death by GCs, with a long-term goal being the specific, targeted intervention in the deregulated pathways and re-sensitization to GCs. For example, there is clear evidence for hyperactivation of AKT [48] and mTOR [25] in GC-resistant B-ALL. As treatment of B-ALL cells with GCs leads to programmed cell death, increased expression of BCL2 family antiapoptotic proteins (e.g. MCL-1) was another particularly interesting finding in GC-resistant B-ALL blasts [18, 25, 49]. As expected, targeting the overexpressed MCL-1 with BH3-mimetic obatoclax increased the cellular sensitivity to GCs; however, the molecular mechanisms leading to resensitization were far more complicated than solely modulation of mitochondrial apoptotic machinery [18, 19, 49]. Obatoclax combined with GC induced multiple cell death pathways (including necroptosis and caspase—dependent and -independent apoptosis), which were preceded by induction of autophagy [18, 19]. Mechanistically, treatment with obatoclax was associated with dissociation of MCL-1 from BCN1 and BAK, increased LC3 processing, LC3 relocalization and decreased p62 abundance [18, 19]. Downregulation of BCN1 or ATG5 decreased the amount of cell death, indicating that autophagy is required for all these cell death mechanisms to occur [18, 19].

These findings are consistent with earlier landmark studies indicating that in B-ALL blasts, autophagy is required for subsequent induction of apoptosis [17]. Formation of autophagosomes occurred early in the sequence of DEX-induced events, before BAK activation, loss of mitochondrial membrane potential and nuclear fragmentation [17]. Furthermore, chemical and genetic inhibition of autophagosome formation led to a decrease in the apoptotic cell death [17]. These data indicate that induction of autophagy by GCs in ALL cells is a prerequisite for subsequent initiation and execution of apoptosis. Mechanistically, induction of autophagy in GC-sensitive cells by dexamethasone involved upregulation of promyelocytic leukemia protein, PML, its complex formation with AKT and a PML-dependent AKT dephosphorylation [17]. Initiation of autophagy and the onset of apoptosis were both dependent on these events. Since AKT is a major regulator of mTOR, thought to play a central role in the regulation of autophagy, these results suggest that DEX-induced autophagy is mediated by the decreased activity of this pathway. Consistent with the key role of autophagy in GCs sensitivity, previous high-throughput studies, utilizing a compendium of drug-induced gene expression signatures and pattern-matching algorithms to identify compounds that modulate the GCs resistance, revealed that rapamycin, an mTORC1 inhibitor, sensitized ALL cells to GCs [25]. Of note, combination of obatoclax with DEX decreased mTOR-AKT activity [19]. GC-resistant ALL cells could therefore be primed for mTOR-controled autophagy.

In this study, using an unbiased bioinformatics approach, we found that GC-resistant ALL cells exhibit significantly higher expression of all MAPK/ERK pathway components (RAS, RAF, MEK1/2, ERK1/2, JNK, cFOS), as compared to sensitive cells, indicating that GCs resistance in ALL cells might be driven by MAPK/ERK pathway signaling. MAPK/ERK activity has been previously implicated in mediating the resistance to GCs in lymphoid cells in several different mechanisms [49–51]. For example, ERK1/2 phosphorylates BIM, which targets this protein for proteasomal degradation [51]. By disrupting this mechanism, MEK inhibitors increase BIM protein level [51]. Other studies indicate that BIM dephosphorylation (resulting from MEK inhibition) allows it to bind to and neutralize MCL-1 [49]. Thus, BIM might compete with MCL1 for BCN1 binding, and released BCN1 might be free to induce autophagy. In more recent studies, MEK2 inhibition was shown to sensitize B-ALL cells to DEX in a p53-dependent manner [50]. Levels of p53 have been previously shown to be modulated through the MEK/ERK pathway via MDM2 [52, 53]. Thus, MEK inhibition in GC-resistant B-ALL cells leads to increased stability and expression of p53 [50]. Introduction of transcriptionally-deficient, dominant-negative p53 allele to cells with siRNA-blocked MEK2 abrogated the resensitization effect, thereby indicating that it requires p53-dependent transcription upon cellular insult [50]. It is particularly interesting, as nuclear (transcriptionally active) pool of p53 is involved in stimulation of autophagy via induction of multiple genes involved in this process [54, 55]. MEK expression and activity in B-ALL cells are also regulated by microRNAs [56]. Low levels of MIR335 are associated with poor outcome in primary pediatric B-ALL, and forced expression of this micro RNA decreased MEK1 expression and increased cellular sensitivity to prednisolone [56].

**Fig 8:**
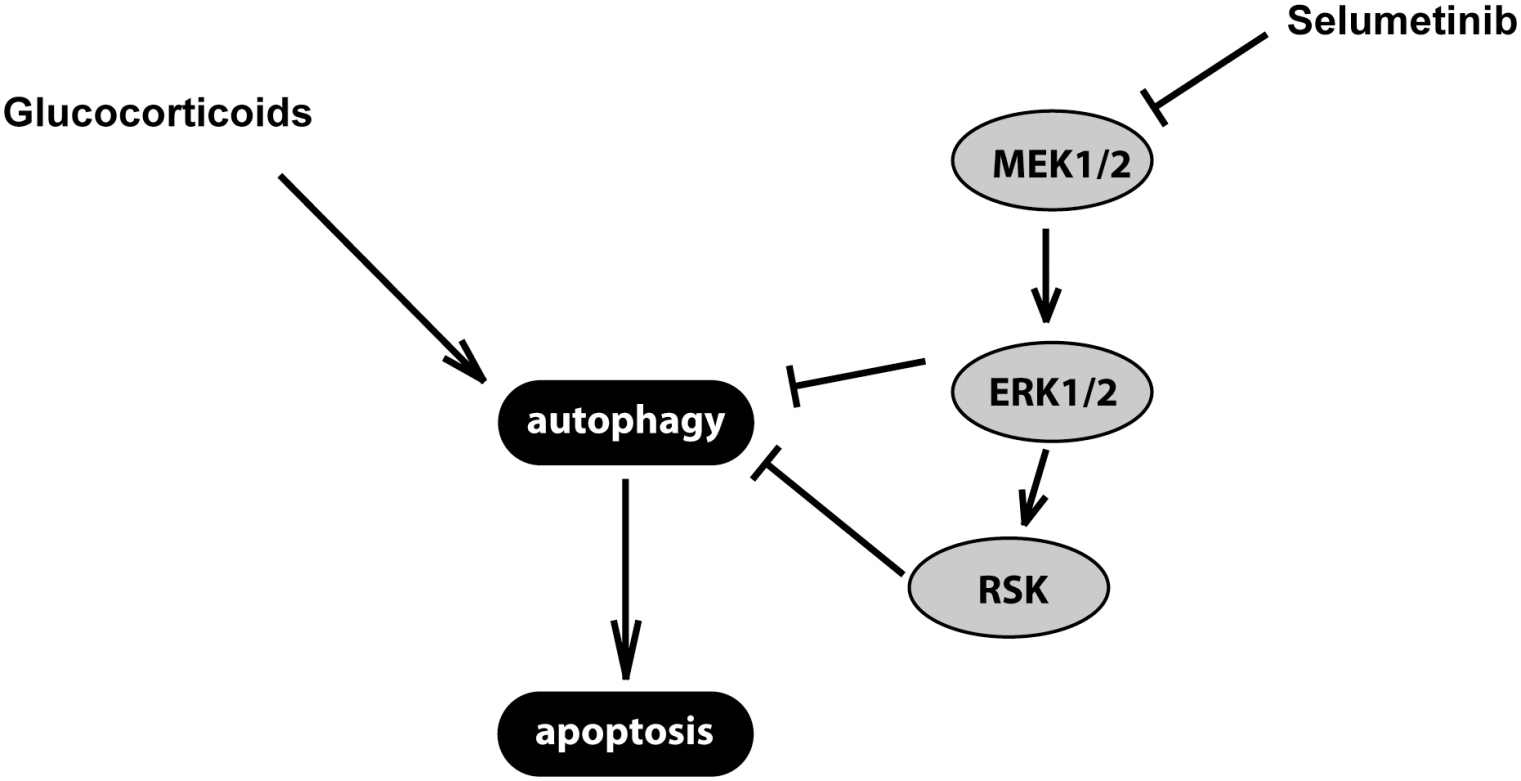
Schematic diagram showing a postulated mechanism of MEK inhibition—induced sensitization to GCs via modulation of autophagy.

These previous studies addressing the mechanisms of MEK1/2 inhibitors-induced sensitization to GCs underscore on the role of apoptotic cell death. Given additional studies, highlighting the role of autophagy upstream of apoptosis [17–19], we hypothesized that MEK/ERK signaling pathway modulation would sensitize DEX-resistant cells in a mechanism involving alterations in autophagy. We demonstrate herein that MEK/ERK1/2 pathway interferes with DEX-mediated induction of cell death by inhibition of mTOR pathway. Mechanistically, these effects are likely mediated by cooperative inhibition of TSC2 complex by AKT and ERK, thereby allowing small GTPase RHEB to activate mTORC1 [45, 57]. Thus, only concurrent blockade of both pathways by GCs and a MEK1/2 inhibitor would allow to block mTORC1 (Fig 8). These changes were associated with increased LC3 processing, LC3 relocalization and MDC staining, which, together, most likely reflect increased autophagy. However, it has to be noted that the nature of these changes cannot be yet unequivocally defined. It is unclear whether the changes in markers of autophagy result from increased autophagy flux and are associated with increased cargo loading. Accumulation of GFP-LC3 puncta can be not only due to induction of autophagy but also to unspecific aggregation of LC3, whereas increased MDC staining can result from the dye’s affinity to certain lipid-rich compartments (autophagic vacuoles and lamellar bodies). Thus, additional studies are required to unequivocally confirm the nature of these changes, to determine the role of autophagy in GC resistance and to demonstrate that MEK inhibition unleashes the potential of DEX to induce autophagy [58].

Altogether, the current and previously published studies highlight complex and multifaceted role of MEK/ERK pathway in modulation of cellular responses to GCs. Although inhibition of p90RSK/TSC2/mTOR by SEL/DEX combination and increased autophagy in this context play an important role, alternative/additional mechanisms operating through altered BIM abundance, MCL1 sequestration and liberation of BCN1 cannot be excluded. Interestingly, in contrast to cytodestructive autophagy triggered by GCs (or by their combinations overcoming GC resistance) in B-ALL blasts, in many solid tumors autophagy is a defense mechanisms, increasing resistance to multiple chemotherapeutics [59, 60]. MEK seems to play an important role in this process, stimulating autophagy via distinct direct mechanisms, such as modulation of BCN1 or by phosphorylating GAIP, which stimulates its GTPase activity towards the GTP-bound conformation of the autophagy-stimulating G_αi3_ protein [61, 62]. Alternatively, Raf/MEK/ERK pathway can affect LC3 levels by affecting its mRNA level, while the MTORC1/RPS6KB1 can affect LC3 protein levels via GRP78/BiP, increasing cellular capacity for autophagy [63]. Furthermore, recent studies indicate that the relationships between ERK and autophagy are bidirectional, as autophagy proteins function as scaffolds regulating ERK phosphorylation and activity [64]. Together, our results further indicate that autophagy is a highly versatile process with composite regulation, highly dependent on cellular context, coexisting signaling circuitry and cellular stressors.

From the clinical standpoint, inhibition of MEK as a strategy to increase GC responses is a very attractive concept, regardless of the underlying mechanism of sensitization. In previous studies, high-level activation of this pathway was present in about 30% of adult ALL patients and was associated with increased WBC at diagnosis and with adverse prognosis [65]. However, unlike in other malignancies, structural abnormalities leading to MEK/ERK activation in B-ALL are uncommon. Activity of MAPK/ERK pathway in B-ALL is also triggered by normal extracellular stimulation through stromal contact and serum growth factors [66]. Treatment with selumetinib resulted in a rapid, complete and persistent reduction of microenvironment-generated pERK1/2. Similarly, in our series of 22 patients, low-level activity of MAPK/ERK was detected in the majority of cases using a similar, sensitive intracellular phospho-specific FACS-based assay. These observations are particularly important, as we found that inhibition of MEK1/2 with selumetinib increased DEX-induced apoptosis in 19 of 22 pERK-positive patients, irrespective of the magnitude of baseline ERK1 activity. Interestingly, the study by George et al [66] revealed a synergy between MEK inhibitors and PI3Kδ inhibitor idelalisib (CAL101), a potent inducer of autophagy in certain B-lineage derived cells [67].

Taken together, we describe the role of MEK/ERK activation in glucocorticoid resistance in ALL cells and characterize a mechanism of MEK1/2 inhibition-induced sensitization to GCs. We show that the mechanism underlying this synergy involves inhibition of mTOR pathway and induction of certain markers of autophagy, likely reflecting induction of this process, leading eventually to cell death. These observations highlight the therapeutic potential of MEK1/2 inhibition in adult ALL and may contribute to the development of targeted therapies increasing the activity of GCs. For example, such inhibitors might be utilized to increase the likelihood of successful reduction in WBC counts in the GC-pretreatment phase or increase chances to achieve a MRD-negative status upon completion of induction phase. Since both these parameters are broadly accepted prognostic factors, such approaches might improve patient outcomes. For these reasons, the place of MEK1/2 inhibitors in the treatment of ALL patients needs to be further evaluated in clinical trials.

## Supporting Information

S1 Fig Phospho-specific flow cytometric analysis of pERK activity in primary ALL blasts obtained from 22 newly diagnosed B-ALL patients. (TIF)

## References

[1] Gaynon PS, Carrel AL. Glucocorticosteroid therapy in childhood acute lymphoblastic leukemia. Adv Exp Med Biol. 1999;457:593–605. . 10500839 10.1007/978-1-4615-4811-9_66

[2] Bassan R. Evolving strategies for the management of high-risk adult acute lymphoblastic leukemia. Haematologica. 2005;90(10):1299 . 16219555

[3] Rowe JM, Buck G, Burnett AK, Chopra R, Wiernik PH, Richards SM, et al Induction therapy for adults with acute lymphoblastic leukemia: results of more than 1500 patients from the international ALL trial: MRC UKALL XII/ECOG E2993. Blood. 2005;106(12):3760–7. 10.1182/blood-2005-04-1623 . 16105981

[4] Maung ZT, Reid MM, Matheson E, Taylor PR, Proctor SJ, Hall AG. Corticosteroid resistance is increased in lymphoblasts from adults compared with children: preliminary results of in vitro drug sensitivity study in adults with acute lymphoblastic leukaemia. Br J Haematol. 1995;91(1):93–100. . 7577660 10.1111/j.1365-2141.1995.tb05251.x

[5] Klumper E, Pieters R, Veerman AJ, Huismans DR, Loonen AH, Hählen K, et al In vitro cellular drug resistance in children with relapsed/refractory acute lymphoblastic leukemia. Blood. 1995;86(10):3861–8. . 7579354

[6] Choi S, Henderson MJ, Kwan E, Beesley AH, Sutton R, Bahar AY, et al Relapse in children with acute lymphoblastic leukemia involving selection of a preexisting drug-resistant subclone. Blood. 2007;110(2):632–9. 10.1182/blood-2007-01-067785 . 17371950

[7] Hongo T, Yajima S, Sakurai M, Horikoshi Y, Hanada R. In vitro drug sensitivity testing can predict induction failure and early relapse of childhood acute lymphoblastic leukemia. Blood. 1997;89(8):2959–65. . 9108416

[8] Kaspers GJ, Pieters R, Van Zantwijk CH, Van Wering ER, Van Der Does-Van Den Berg A, Veerman AJ. Prednisolone resistance in childhood acute lymphoblastic leukemia: vitro-vivo correlations and cross-resistance to other drugs. Blood. 1998;92(1):259–66. . 9639525

[9] Dhédin N, Huynh A, Maury S, Tabrizi R, Beldjord K, Asnafi V, et al Role of allogeneic stem cell transplantation in adult patients with Ph-negative acute lymphoblastic leukemia. Blood. 2015;125(16):2486–96; quiz 586. 10.1182/blood-2014-09-599894 . 25587040

[10] Consolaro F, Ghaem-Maghami S, Bortolozzi R, Zona S, Khongkow M, Basso G, et al FOXO3a and Posttranslational Modifications Mediate Glucocorticoid Sensitivity in B-ALL. Mol Cancer Res. 2015;13(12):1578–90. Epub 2015/09/18. 10.1158/1541-7786.MCR-15-0127 1541-7786.MCR-15-0127 [pii]. . 26376801

[11] Zhou M, Wang T, Lai H, Zhao X, Yu Q, Zhou J, et al Targeting of the deubiquitinase USP9X attenuates B-cell acute lymphoblastic leukemia cell survival and overcomes glucocorticoid resistance. Biochem Biophys Res Commun. 2015;459(2):333–9. Epub 2015/03/05. 10.1016/j.bbrc.2015.02.115 S0006-291X(15)00355-1 [pii]. . 25735983

[12] Kfir-Erenfeld S, Sionov RV, Spokoini R, Cohen O, Yefenof E. Protein kinase networks regulating glucocorticoid-induced apoptosis of hematopoietic cancer cells: fundamental aspects and practical considerations. Leuk Lymphoma. 2010;51(11):1968–2005. 10.3109/10428194.2010.506570 . 20849387

[13] Sionov RV, Spokoini R, Kfir-Erenfeld S, Cohen O, Yefenof E. Mechanisms regulating the susceptibility of hematopoietic malignancies to glucocorticoid-induced apoptosis. Adv Cancer Res. 2008;101:127–248. 19055945 10.1016/S0065-230X(08)00406-5

[14] Spokoini R, Kfir-Erenfeld S, Yefenof E, Sionov RV. Glycogen synthase kinase-3 plays a central role in mediating glucocorticoid-induced apoptosis. Mol Endocrinol. 2010;24(6):1136–50. 10.1210/me.2009-0466 . 20371704 PMC5417474

[15] Distelhorst CW. Recent insights into the mechanism of glucocorticosteroid-induced apoptosis. Cell Death Differ. 2002;9(1):6–19. 10.1038/sj.cdd.4400969 . 11803370

[16] Wang Z, Malone MH, He H, McColl KS, Distelhorst CW. Microarray analysis uncovers the induction of the proapoptotic BH3-only protein Bim in multiple models of glucocorticoid-induced apoptosis. J Biol Chem. 2003;278(26):23861–7. 10.1074/jbc.M301843200 . 12676946

[17] Laane E, Tamm KP, Buentke E, Ito K, Kharaziha P, Khahariza P, et al Cell death induced by dexamethasone in lymphoid leukemia is mediated through initiation of autophagy. Cell Death Differ. 2009;16(7):1018–29. 10.1038/cdd.2009.46 . 19390558

[18] Heidari N, Hicks MA, Harada H. GX15-070 (obatoclax) overcomes glucocorticoid resistance in acute lymphoblastic leukemia through induction of apoptosis and autophagy. Cell Death Dis. 2010;1:e76 Epub 2011/03/03. 10.1038/cddis.2010.53 cddis201053 [pii]. 21364679 PMC3032343

[19] Bonapace L, Bornhauser BC, Schmitz M, Cario G, Ziegler U, Niggli FK, et al Induction of autophagy-dependent necroptosis is required for childhood acute lymphoblastic leukemia cells to overcome glucocorticoid resistance. J Clin Invest. 2010;120(4):1310–23. 10.1172/jci39987 20200450 PMC2846044

[20] Feng Y, He D, Yao Z, Klionsky DJ. The machinery of macroautophagy. Cell Res. 2014;24(1):24–41. 10.1038/cr.2013.168 24366339 PMC3879710

[21] Petiot A, Ogier-Denis E, Blommaart EF, Meijer AJ, Codogno P. Distinct classes of phosphatidylinositol 3'-kinases are involved in signaling pathways that control macroautophagy in HT-29 cells. J Biol Chem. 2000;275(2):992–8. . 10625637 10.1074/jbc.275.2.992

[22] Tassa A, Roux MP, Attaix D, Bechet DM. Class III phosphoinositide 3-kinase—Beclin1 complex mediates the amino acid-dependent regulation of autophagy in C2C12 myotubes. Biochem J. 2003;376(Pt 3):577–86. Epub 2003/09/12. 10.1042/BJ20030826 BJ20030826 [pii]. 12967324 PMC1223813

[23] Gozuacik D, Kimchi A. Autophagy as a cell death and tumor suppressor mechanism. Oncogene. 2004;23(16):2891–906. 10.1038/sj.onc.1207521 . 15077152

[24] Gozuacik D, Kimchi A. Autophagy and cell death. Curr Top Dev Biol. 2007;78:217–45. 10.1016/s0070-2153(06)78006-1 . 17338918

[25] Wei G, Twomey D, Lamb J, Schlis K, Agarwal J, Stam RW, et al Gene expression-based chemical genomics identifies rapamycin as a modulator of MCL1 and glucocorticoid resistance. Cancer Cell. 2006;10(4):331–42. 10.1016/j.ccr.2006.09.006 . 17010674

[26] Spijkers-Hagelstein JA, Schneider P, Hulleman E, de Boer J, Williams O, Pieters R, et al Elevated S100A8/S100A9 expression causes glucocorticoid resistance in MLL-rearranged infant acute lymphoblastic leukemia. Leukemia. 2012;26(6):1255–65. 10.1038/leu.2011.388 . 22282267

[27] Golub TR, Slonim DK, Tamayo P, Huard C, Gaasenbeek M, Mesirov JP, et al Molecular classification of cancer: class discovery and class prediction by gene expression monitoring. Science. 1999;286(5439):531–7. . 10521349 10.1126/science.286.5439.531

[28] Kim C, Cheon M, Kang M, Chang I. A simple and exact Laplacian clustering of complex networking phenomena: application to gene expression profiles. Proc Natl Acad Sci U S A. 2008;105(11):4083–7. 10.1073/pnas.0708598105 18337496 PMC2393820

[29] Lamb J, Crawford ED, Peck D, Modell JW, Blat IC, Wrobel MJ, et al The Connectivity Map: using gene-expression signatures to connect small molecules, genes, and disease. Science. 2006;313(5795):1929–35. 10.1126/science.1132939 . 17008526

[30] Benjamini Y, Drai D, Elmer G, Kafkafi N, Golani I. Controlling the false discovery rate in behavior genetics research. Behav Brain Res. 2001;125(1–2):279–84. . 11682119 10.1016/s0166-4328(01)00297-2

[31] Polo JM, Juszczynski P, Monti S, Cerchietti L, Ye K, Greally JM, et al Transcriptional signature with differential expression of BCL6 target genes accurately identifies BCL6-dependent diffuse large B cell lymphomas. Proc Natl Acad Sci U S A. 2007;104(9):3207–12. 10.1073/pnas.0611399104 17360630 PMC1805543

[32] Subramanian A, Tamayo P, Mootha VK, Mukherjee S, Ebert BL, Gillette MA, et al Gene set enrichment analysis: a knowledge-based approach for interpreting genome-wide expression profiles. Proc Natl Acad Sci U S A. 2005;102(43):15545–50. 10.1073/pnas.0506580102 16199517 PMC1239896

[33] Morgenstern JP, Land H. Advanced mammalian gene transfer: high titre retroviral vectors with multiple drug selection markers and a complementary helper-free packaging cell line. Nucleic Acids Res. 1990;18(12):3587–96. 2194165 10.1093/nar/18.12.3587 PMC331014

[34] Boehm JS, Zhao JJ, Yao J, Kim SY, Firestein R, Dunn IF, et al Integrative genomic approaches identify IKBKE as a breast cancer oncogene. Cell. 2007;129(6):1065–79. 10.1016/j.cell.2007.03.052 . 17574021

[35] Kabeya Y, Mizushima N, Ueno T, Yamamoto A, Kirisako T, Noda T, et al LC3, a mammalian homologue of yeast Apg8p, is localized in autophagosome membranes after processing. EMBO J. 2000;19(21):5720–8. 10.1093/emboj/19.21.5720 11060023 PMC305793

[36] Juszczynski P, Kutok JL, Li C, Mitra J, Aguiar RC, Shipp MA. BAL1 and BBAP are regulated by a gamma interferon-responsive bidirectional promoter and are overexpressed in diffuse large B-cell lymphomas with a prominent inflammatory infiltrate. Mol Cell Biol. 2006;26(14):5348–59. 10.1128/mcb.02351-05 16809771 PMC1592708

[37] Abramson JS, Chen W, Juszczynski P, Takahashi H, Neuberg D, Kutok JL, et al The heat shock protein 90 inhibitor IPI-504 induces apoptosis of AKT-dependent diffuse large B-cell lymphomas. Br J Haematol. 2009;144(3):358–66. 10.1111/j.1365-2141.2008.07484.x 19036086 PMC4029164

[38] Juszczynski P, Chen L, O'Donnell E, Polo JM, Ranuncolo SM, Dalla-Favera R, et al BCL6 modulates tonic BCR signaling in diffuse large B-cell lymphomas by repressing the SYK phosphatase, PTPROt. Blood. 2009;114(26):5315–21. 10.1182/blood-2009-02-204362 19855081 PMC2796136

[39] Emery CM, Vijayendran KG, Zipser MC, Sawyer AM, Niu L, Kim JJ, et al MEK1 mutations confer resistance to MEK and B-RAF inhibition. Proc Natl Acad Sci U S A. 2009;106(48):20411–6. 10.1073/pnas.0905833106 19915144 PMC2777185

[40] Mizushima N, Yoshimori T. How to interpret LC3 immunoblotting. Autophagy. 2007;3(6):542–5. . 17611390 10.4161/auto.4600

[41] Zhang XJ, Chen S, Huang KX, Le WD. Why should autophagic flux be assessed? Acta Pharmacol Sin. 2013;34(5):595–9. 10.1038/aps.2012.184 23474710 PMC4002868

[42] Klionsky DJ, Abdalla FC, Abeliovich H, Abraham RT, Acevedo-Arozena A, Adeli K, et al Guidelines for the use and interpretation of assays for monitoring autophagy. Autophagy. 2012;8(4):445–544. 22966490 10.4161/auto.19496 PMC3404883

[43] Ni HM, Bockus A, Wozniak AL, Jones K, Weinman S, Yin XM, et al Dissecting the dynamic turnover of GFP-LC3 in the autolysosome. Autophagy. 2011;7(2):188–204. 21107021 10.4161/auto.7.2.14181 PMC3039769

[44] Kim YC, Guan KL. mTOR: a pharmacologic target for autophagy regulation. J Clin Invest. 2015;125(1):25–32. 10.1172/jci73939 25654547 PMC4382265

[45] Ma L, Teruya-Feldstein J, Bonner P, Bernardi R, Franz DN, Witte D, et al Identification of S664 TSC2 phosphorylation as a marker for extracellular signal-regulated kinase mediated mTOR activation in tuberous sclerosis and human cancer. Cancer Res. 2007;67(15):7106–12. 10.1158/0008-5472.can-06-4798 . 17671177

[46] Anjum R, Blenis J. The RSK family of kinases: emerging roles in cellular signalling. Nat Rev Mol Cell Biol. 2008;9(10):747–58. 10.1038/nrm2509 . 18813292

[47] Roux PP, Ballif BA, Anjum R, Gygi SP, Blenis J. Tumor-promoting phorbol esters and activated Ras inactivate the tuberous sclerosis tumor suppressor complex via p90 ribosomal S6 kinase. Proc Natl Acad Sci U S A. 2004;101(37):13489–94. 10.1073/pnas.0405659101 15342917 PMC518784

[48] Bornhauser BC, Bonapace L, Lindholm D, Martinez R, Cario G, Schrappe M, et al Low-dose arsenic trioxide sensitizes glucocorticoid-resistant acute lymphoblastic leukemia cells to dexamethasone via an Akt-dependent pathway. Blood. 2007;110(6):2084–91. Epub 2007/06/01. blood-2006-12-060970 [pii] 10.1182/blood-2006-12-060970 . 17537996

[49] Korfi K, Smith M, Swan J, Somervaille TC, Dhomen N, Marais R. BIM mediates synergistic killing of B-cell acute lymphoblastic leukemia cells by BCL-2 and MEK inhibitors. Cell Death Dis. 2016;7:e2177 Epub 2016/04/08. 10.1038/cddis.2016.70 cddis201670 [pii]. . 27054332 PMC4855656

[50] Jones CL, Gearheart CM, Fosmire S, Delgado-Martin C, Evensen NA, Bride K, et al MAPK signaling cascades mediate distinct glucocorticoid resistance mechanisms in pediatric leukemia. Blood. 2015;126(19):2202–12. Epub 2015/09/02. 10.1182/blood-2015-04-639138 blood-2015-04-639138 [pii]. . 26324703 PMC4635116

[51] Rambal AA, Panaguiton ZL, Kramer L, Grant S, Harada H. MEK inhibitors potentiate dexamethasone lethality in acute lymphoblastic leukemia cells through the pro-apoptotic molecule BIM. Leukemia. 2009;23(10):1744–54. 10.1038/leu.2009.80 19404317 PMC2761998

[52] Ries S, Biederer C, Woods D, Shifman O, Shirasawa S, Sasazuki T, et al Opposing effects of Ras on p53: transcriptional activation of mdm2 and induction of p19ARF. Cell. 2000;103(2):321–30. Epub 2000/11/01. S0092-8674(00)00123-9 [pii]. . 11057904 10.1016/s0092-8674(00)00123-9

[53] Phelps M, Phillips A, Darley M, Blaydes JP. MEK-ERK signaling controls Hdm2 oncoprotein expression by regulating hdm2 mRNA export to the cytoplasm. J Biol Chem. 2005;280(17):16651–8. Epub 2005/02/23. M412334200 [pii] 10.1074/jbc.M412334200 . 15723837

[54] Maiuri MC, Galluzzi L, Morselli E, Kepp O, Malik SA, Kroemer G. Autophagy regulation by p53. Curr Opin Cell Biol. 2010;22(2):181–5. Epub 2010/01/02. 10.1016/j.ceb.2009.12.001 S0955-0674(09)00230-0 [pii]. . 20044243

[55] Pietrocola F, Izzo V, Niso-Santano M, Vacchelli E, Galluzzi L, Maiuri MC, et al Regulation of autophagy by stress-responsive transcription factors. Semin Cancer Biol. 2013;23(5):310–22. Epub 2013/06/04. 10.1016/j.semcancer.2013.05.008 S1044-579X(13)00050-3 [pii]. . 23726895

[56] Yan J, Jiang N, Huang G, Tay JL, Lin B, Bi C, et al Deregulated MIR335 that targets MAPK1 is implicated in poor outcome of paediatric acute lymphoblastic leukaemia. Br J Haematol. 2013;163(1):93–103. Epub 2013/07/31. 10.1111/bjh.12489 . 23888996

[57] Huang J, Manning BD. A complex interplay between Akt, TSC2 and the two mTOR complexes. Biochem Soc Trans. 2009;37(Pt 1):217–22. 10.1042/bst0370217 19143635 PMC2778026

[58] Klionsky DJ, Abdelmohsen K, Abe A, Abedin MJ, Abeliovich H, Acevedo Arozena A, et al Guidelines for the use and interpretation of assays for monitoring autophagy (3rd edition). Autophagy. 2016;12(1):1–222. Epub 2016/01/23. 10.1080/15548627.2015.1100356 26799652 PMC4835977

[59] Yang ZJ, Chee CE, Huang S, Sinicrope FA. The role of autophagy in cancer: therapeutic implications. Mol Cancer Ther. 2011;10(9):1533–41. Epub 2011/09/01. 10.1158/1535-7163.MCT-11-0047 1535-7163.MCT-11-0047 [pii]. 21878654 PMC3170456

[60] Mirzoeva OK, Hann B, Hom YK, Debnath J, Aftab D, Shokat K, et al Autophagy suppression promotes apoptotic cell death in response to inhibition of the PI3K-mTOR pathway in pancreatic adenocarcinoma. J Mol Med (Berl). 2011;89(9):877–89. Epub 2011/06/17. 10.1007/s00109-011-0774-y . 21678117

[61] Wang J, Whiteman MW, Lian H, Wang G, Singh A, Huang D, et al A non-canonical MEK/ERK signaling pathway regulates autophagy via regulating Beclin 1. J Biol Chem. 2009;284(32):21412–24. Epub 2009/06/13. 10.1074/jbc.M109.026013 M109.026013 [pii]. 19520853 PMC2755866

[62] Ogier-Denis E, Pattingre S, El Benna J, Codogno P. Erk1/2-dependent phosphorylation of Galpha-interacting protein stimulates its GTPase accelerating activity and autophagy in human colon cancer cells. J Biol Chem. 2000;275(50):39090–5. Epub 2000/09/20. 10.1074/jbc.M006198200 M006198200 [pii]. . 10993892

[63] Kim JH, Hong SK, Wu PK, Richards AL, Jackson WT, Park JI. Raf/MEK/ERK can regulate cellular levels of LC3B and SQSTM1/p62 at expression levels. Exp Cell Res. 2014;327(2):340–52. Epub 2014/08/17. 10.1016/j.yexcr.2014.08.001 S0014-4827(14)00329-2 [pii]. 25128814 PMC4164593

[64] Martinez-Lopez N, Athonvarangkul D, Mishall P, Sahu S, Singh R. Autophagy proteins regulate ERK phosphorylation. Nat Commun. 2013;4:2799 Epub 2013/11/19. 10.1038/ncomms3799 ncomms3799 [pii]. 24240988 PMC3868163

[65] Gregorj C, Ricciardi MR, Petrucci MT, Scerpa MC, De Cave F, Fazi P, et al ERK1/2 phosphorylation is an independent predictor of complete remission in newly diagnosed adult acute lymphoblastic leukemia. Blood. 2007;109(12):5473–6. 10.1182/blood-2006-05-021071 . 17351113

[66] George AA, Paz H, Fei F, Kirzner J, Kim YM, Heisterkamp N, et al Phosphoflow-Based Evaluation of Mek Inhibitors as Small-Molecule Therapeutics for B-Cell Precursor Acute Lymphoblastic Leukemia. PLoS One. 2015;10(9):e0137917 Epub 2015/09/12. 10.1371/journal.pone.0137917 PONE-D-15-18997 [pii]. 26360058 PMC4567297

[67] Ikeda H, Hideshima T, Fulciniti M, Perrone G, Miura N, Yasui H, et al PI3K/p110{delta} is a novel therapeutic target in multiple myeloma. Blood. 2010;116(9):1460–8. Epub 2010/05/28. 10.1182/blood-2009-06-222943 blood-2009-06-222943 [pii]. 20505158 PMC2938837

