# Supplementary figures and images for: MEK Inhibition Sensitizes Precursor B-Cell Acute Lymphoblastic Leukemia (B-ALL) Cells to Dexamethasone through Modulation of mTOR Activity and Stimulation of Autophagy
Source: PLoS One. 2016 May 19;11(5):e0155893. doi: 10.1371/journal.pone.0155893 (PMC4872998; doi:10.1371/journal.pone.0155893)

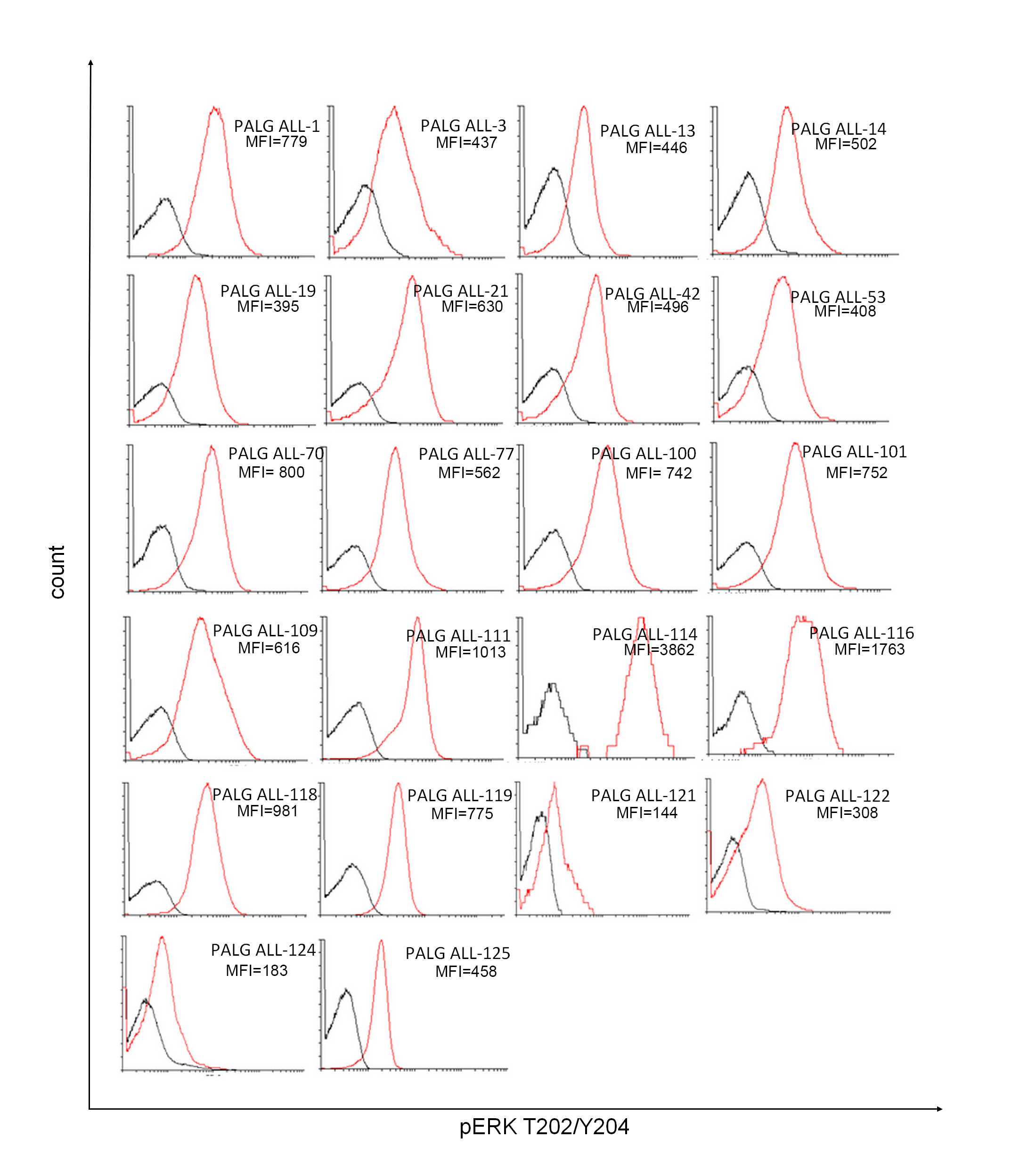

Supplement: S1 Fig — (TIF) [file pone.0155893.s001.tif]
